# Public health implications of recent declines in fatal drug overdoses in New York State and the United States

**DOI:** 10.1093/haschl/qxae172

**Published:** 2024-12-12

**Authors:** David R Holtgrave, Allan Clear, James V McDonald

**Affiliations:** New York State Department of Health, Office of the Commissioner, Albany, NY 12237, United States; Department of Health, Behavior and Society (adjunct), Johns Hopkins Bloomberg School of Public Health, Baltimore, MD 21205, United States; New York State Department of Health, AIDS Institute, Office of Drug User Health, Albany, NY 12237, United States; New York State Department of Health, Office of the Commissioner, Albany, NY 12237, United States

**Keywords:** substance-use policy, public health policy, drug overdose, policy planning

## Introduction

After a period of substantial increases in fatal drug overdoses in New York State and the United States, the Centers for Disease Control and Prevention (CDC) provisional data (as of October 16, 2024) indicate a decline of 12.33% in New York and 12.71% in the United States for the 12-month period ending May 2024 relative to the year ending May 2023.^1^ However, timely policy-relevant questions^2^ remaining to be answered include the following: If fatal overdose trends from 2019 through 2023 had continued into 2024, what would the projected number of drug overdose deaths have been? How many lives were saved if one compares CDC provisional fatal overdose data with a projected counterfactual for 2024? Besides lives saved, what do the deaths averted mean in terms of total years of life saved? Using a standard regulatory analysis framework, what is the value of statistical lives saved as a function of recent declines in fatal overdoses? Do the recent declines indicate whether the national 2025 fatal overdose goal is within reach?

## Methods

Besides directly comparing year-ending May 2024 with year-ending May 2023 fatal drug overdose data to calculate fatalities averted,^1^ a counterfactual was estimated using linear forecasting from the 5 years 2019 to 2023 (all ending in May of the respective year) to estimate the possible value of fatal overdoses for the 12-month period ending in May 2024 if prior trends had continued.^1,3^ This yields 2 estimates of the number of fatalities averted for New York and the United States (year-over-year “direct comparison” and comparison to a calculated counterfactual).

Both a direct comparison and a comparison to the counterfactual approach were used to estimate the impact of fatal overdose declines on years of life saved (YLS). To estimate the undiscounted YLS for 1 fatal overdose averted, the average age of death due to fatal overdose in the nation was calculated using data by age group from CDC's SUDORS (State Unintentional Drug Overdose Reporting System) dashboard^4^ and comparing that result with CDC's FastStat life expectancy data^5^ (after adjusting for sex). The number of YLS for each drug-overdose fatality avoided was then multiplied by the number of deaths averted to yield an estimate of overall YLS.

To estimate the value of statistical lives (VSL) saved by the drug-overdose fatalities averted, a similar direct comparison and comparison to the counterfactual approach was used. For the value of 1 statistical life saved, low, medium, and high values of $6.1 million, $13.0 million, and $19.7 million (2023 dollars) were taken from standard values recommended by the Department of Health and Human Services for regulatory analyses.^6^

The National Drug Control Strategy goal for reducing fatal drug overdoses is (no more than) 90 000 for the year 2024 and 81 000 for 2025.^7^ To estimate whether these goals may be met, recent percentage declines in fatal overdoses were applied to year-ending December 2023 CDC provisional data to construct projections of year-ending December 2024 and 2025 fatal overdoses.

## Results

**Figure 1.**
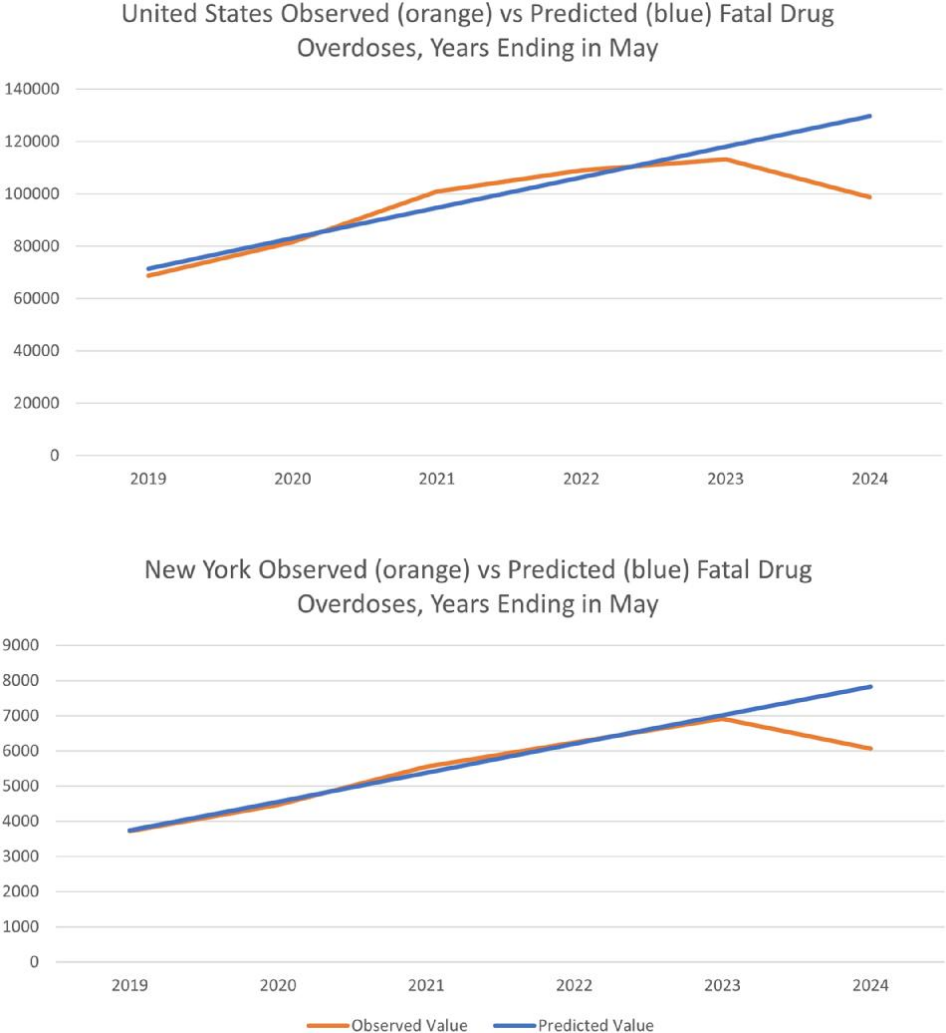
Provisional observed vs predicted fatal drug overdoses for years ending in May (United States and New York State).

Linear regression provided a good fit to the data from 2019 through 2023 (details are provided in the Table 1 footnote and graphical representations are given in Figure 1). The fit was so strong that other functional form representations are not provided for the sake of parsimony.

**Table 1.** Implications of declines in fatal drug overdoses for the 12-month period ending May 2024 on deaths averted, years of life saved, and economic value of statistical lives saved.

Result domain	United States	New York State
Reported provisional value of fatal drug overdoses for 12-month period ending May 2024^a1^	98 820	6067
Forecasted counterfactual of fatal drug overdoses for 12-month period ending May 2024 (linear forecasting from data for 5 prior years each ending in May)^a^	129 650	7835
Decrease in fatal overdoses for 12-month period ending May 2024 relative to 12-month period ending May 2023	14 391	853
Decrease in fatal overdoses for 12-month period ending May 2024 relative to forecasted counterfactual	30 830	1768
Years of life saved by fatal overdoses averted in 12-month period ending May 2024 relative to 12-month period ending May 2023	472 493	28 006
Years of life saved by fatal overdoses averted in 12-month period ending May 2024 relative to forecasted counterfactual	1 012 239	58 038
Economic value of statistical lives saved by fatal overdoses averted in 12-month period ending May 2024 relative to 12-month period ending May 2023		
Low	$87 785 100 000	$5 203 300 000
Medium	$187 083 000 000	$11 089 000 000
High	$283 502 700 000	$16 804 100 000
Economic value of statistical lives saved by fatal overdoses averted in 12-month period ending May 2024 relative to forecasted counterfactual		
Low	$188 065 440 000	$10 782 970 000
Medium	$400 795 200 000	$22 980 100 000
High	$607 358 880 000	$34 823 690 000

^a^The correlation squared (ie, coefficient of determination) of Centers for Disease Control and Prevention (CDC)–reported provisional data for the years 2019 through 2023 each ending in May with the linear regression estimates for the same time periods was 0.945 for the nation and 0.992 for New York, indicating that a straight line provided a good fit to the data; *P* < .01 in both cases, with *F* = 51.36 on 1 and 3 degrees of freedom for the United States and *F* = 388.03 on 1 and 3 degrees of freedom for New York State. The estimated regression parameters from those 5 years were used to calculate the forecasted counterfactual provided here for the year ending May 2024.

Had the 2019 through 2023 trends continued into 2024 (estimated from the linear regression equations), there would have been 129 650 fatal overdoses in the United States (rather than the 98 820 that were provisionally estimated by CDC for the year ending May 2024), and there would have been 7835 fatal overdoses in New York (rather than 6067 provisionally estimated by CDC for the year ending May 2024). This more than doubles the number of estimated fatalities averted obtained by simply comparing year-ending May 2024 with year-ending May 2023 fatalities averted. The YLS for the United States ranges from 472 493 to over 1 million. For New York, YLS ranged from over 28 000 to over 58 000.

The economic VSL saved for the United States ranges from approximately $87.785 billion to over $607 billion; for New York, the VSL-saved estimates range from over $5.203 billion to over $34.823 billion.

Applying recent percentage declines in fatal drug overdoses to CDC provisional data for the year ending December 2023 (108 323^1^) projects that year-ending December 2024 fatal overdoses may be approximately 94 553 and for year-ending December 2025 may be approximately 82 534.

## Discussion

The impact on lives, YLS, and VSL saved of recent fatal overdose declines is clearly substantial. Further, the continuation of recent decreases suggests that the national goal for fatal overdoses might be achieved with sufficient scaling up of effective programs and policies. This would be a key milestone toward ultimately achieving a zero-overdose generation.

## Supplementary Material

qxae172_Supplementary_Data
